# Death of Dr. J. R. Hamill

**Published:** 1870-01

**Authors:** B. T. Buckly, Louis Stoskopf

**Affiliations:** President; Secretary


Died — At Davis, Stephenson Co., Ills., on the 7th of October ult., Dr. J. R. Hamill. The deceased was born in Baldwinsville, Onondaga Co., N. Y., March 1, 1828. Read medicine with his brother, A. P. Hamill, and graduated at Geneva. He moved to Stephenson Co. in the year of 1856, and located at Davis, where he was engaged in the practice of his profession until within one week of his death.

Autopsy revealed the cause of his death to be acute inflammation, affecting, principally, the peritoneum duodenum and biliary ducts, caused by biliary obstruction. A large number of gall-stone were found impacted in the common duct, confirming the diagnosis of his attending physicians.

At a special meeting of the Stephenson Co. Medical Society, Oct. 11, 1869, convened for the purpose of taking appropriate action on the death of J. R. Hamill of Davis, the following preamble and resolutions were adopted.

Whereas, It has pleased Almighty God, suddenly to remove from among us Dr. J. R. Hamill, in the vigor of his manhood and in the midst of a career of usefulness;

*Resolved*, That while we bow with humble submission to the will of Him who doeth all things well, we mourn with sincere and heartfelt sorrow the untimely death of a beloved and honored associate.

*Resolved*, That in our deep affliction, we refer with melancholy pride to the many excellent qualities of mind, and heart of Dr. Hamill, which rendered him a welcome and trustworthy counselor to his professional associates, and a valued friend and adviser to the sick and afflicted. As a physician he was gifted with swift and unerring judgment, rich and varied attainments, deep sympathy for the afflicted, and a profound knowledge of the resources of our art. Untiring in the discharge of his professional duties, he was alike ready to obey the summons of the rich and the distressed calls of the poor and needy. Firm in his convictions, self-reliant, and tenacious of his opinions, he was nevertheless always mindful of the rights and feelings of others. As a man he was generous and just; as a physician zealous and intelligent; as a husband and father, kind and indulgent.

*Resolved*, That in his death our Society has lost an earnest and valuable member, and our profession an honored and devoted associate.

*Resolved*, That as fellow-sufferers we tender our sincere sympathies to the afflicted wife and children of the deceased.

*Resolved*, That a copy of these resolutions be inscribed on the records of this society, and that copies be transmitted to the papers of Stephenson County, and to the Chicago Medical Journal, for publication. B. T. Buckly, President.

Louis Stoskopf, Secretary

